# Modulation of cabozantinib efficacy by the prostate tumor microenvironment

**DOI:** 10.18632/oncotarget.21248

**Published:** 2017-09-23

**Authors:** Manisha Tripathi, Srinivas Nandana, Sandrine Billet, Karen A. Cavassani, Rajeev Mishra, Leland W.K. Chung, Edwin M. Posadas, Neil A. Bhowmick

**Affiliations:** ^1^ Department of Medicine, Cedars-Sinai Medical Center, Los Angeles, California 90048, USA; ^2^ Department of Research, Greater Los Angeles Veterans Administration, Los Angeles, California 90048, USA

**Keywords:** carcinoma associated fibroblasts, cabozantinib, metastasis, prostate cancer

## Abstract

The tumor microenvironment (TME) is increasingly recognized as the arbiter of metastatic progression and drug resistance in advanced prostate cancer (PCa). Cabozantinib is a potent tyrosine kinase inhibitor (TKI) with reported biological activity in the PCa epithelia, but failed to provide an overall survival benefit in phase 3 clinical trials. However, the promising biologic efficacy of the drug in early trials warranted a better understanding of the mechanism of action, with the goal of improving patient selection for TKI-based therapy such as cabozantinib. We found a 100-fold lower cabozantinib IC_50_ in macrophages, PCa associated fibroblasts, and bone marrow fibroblasts compared to PCa epithelia. In PCa mouse models, pre-treatment with cabozantinib potentiated osseous and visceral tumor engraftment, suggesting a pro-tumorigenic host response to the drug. We further found that the host effects of cabozantinib impacted bone turnover, but not necessarily tumor expansion. Cabozantinib affected M1 macrophage polarization in mice. Analogously, circulating monocytes from PCa patients treated with cabozantinib, demonstrated a striking correlation of monocyte reprograming with therapeutic bone responsivity, to support patient selection at early stages of treatment. Thus, a re-evaluation of TKI-based therapeutic strategies in PCa can be considered for suitable patient populations based on TME responses.

## INTRODUCTION

Prostate cancer (PCa) is the most common non-cutaneous malignancy and the second leading cause of male cancer death in the western world. Despite advances in local therapy, 27,000 men die of metastatic disease in the US every year. In the metastatic disease setting, androgen deprivation and taxane-based therapy provide initial benefit, but invariably the disease progresses to an intractable metastatic castration-resistant PCa (mCRPC) state.

Tyrosine kinase inhibitors (TKIs) exploit the vulnerability of cells with mutated, overexpressed, and/or activated oncogenes. However, as with most TKI strategies, tumor heterogeneity and therapeutic-induced genetic drift can diminish the efficacy of the anti-tumor effect, contributing to the development of resistance [1]. The activation of a set of kinases including MET and VEGFR in mCRPC epithelial cells and other tumor models drove interest in cabozantinib (XL184, Cometriq, Cabometyx). Cabozantinib is approved for treatment of medullary thyroid and advanced kidney cancers. It has additionally been evaluated for a range of solid tumors including breast, ovarian, lung, skin, and liver cancers as well as mCRPC [2–7].

Pre-clinical studies utilizing PCa animal models showed that cabozantinib inhibits tumor proliferation and bone resorption suggesting that cabozantinib affects both the tumor and bone microenvironments [8, 9]. Moreover, Phase II studies of cabozantinib in CRPC patients identified clinical improvement (*i.e.* pain reduction) accompanied with improvements on radionuclide bone scans [10–13]. These encouraging results led to randomized placebo-controlled phase 3 trials (COMET-1, COMET-2) powered to measure overall survival and pain response. Although cabozantinib did not increase the overall survival, the COMET-1 study identified improvement in radiographic progression free survival (5.6 vs. 2.8 months HR 0.48) and bone scan response (42% vs. 3%) in men previously treated with docetaxel and abiraterone and/or enzalutamide, in the context of favorable circulating tumor cell (CTC) conversions [14]. For metastatic renal cell carcinoma, there was an observed reduction of skeletal related events with cabozantinib treatment in a Phase III study (METEOR) associated with a reduced risk of disease progression and death compared with everolimus [15–17].

Overall, these clinical and pre-clinical studies lend credence to the hypothesis that the bone microenvironment is a potential mediator of cabozantinib efficacy in metastatic bone disease, including mCRPC. This study was undertaken to explore the cabozantinib response of fibroblastic cells and macrophages in PCa progression. TKI’s are capable of inducing immunogenic modulation of macrophages [18]. For simplicity, lipopolysaccharide (LPS) and/or interferon gamma activated macrophages (M1) play a critical role in host defense and anti-tumor immunity [19]. Alternatively, macrophages activated by IL-4/IL-13 (M2) promote wound healing and show pro-tumor activity [19]. However, these are extreme states in a spectrum of macrophage activation and polarity observed *in vivo*. We identified a putative biomarker to identify patients who are likely to have an improvement in their PCa osseous metastatic response, based on cabozantinib-mediated reprogramming of circulating monocytes.

## RESULTS

### Differential effect of cabozantinib on prostate tumors and host

**Figure 1:**
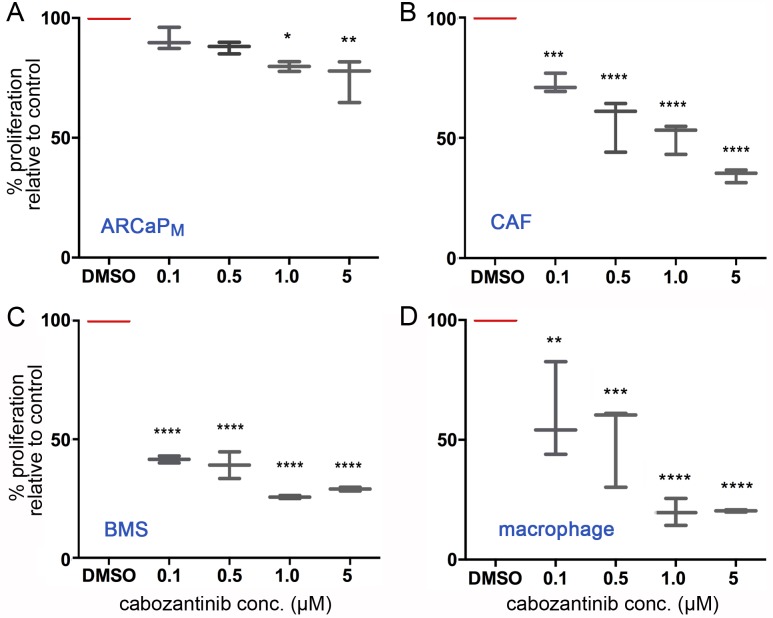
Cabozantinib has a greater efficacy on the tumor microenvironment compared with PCa cells **(A)** ARCaP_M_, **(B)** CAF, **(C)** bone marrow stromal cells (BMS), and **(D)** macrophage were treated with indicated concentrations of cabozantinib for 72 hours, followed by cell viability detection by MTT-assay. Vehicle (DMSO) served as the control. Statistical significance was determined using one-way ANOVA analysis with Dunnett's multiple comparisons test (^*^p value ≤ 0.05, ^**^p value ≤ 0.01, ^***^p value ≤ 0.001, ^****^p value ≤ 0.0001).

To test the direct role of cabozantinib on the proliferative potential of PCa epithelia and host cells, we performed MTT assays. Over a dose range of cabozantinib 0 – 5 μM, no significant proliferative change in ARCaP_M_ epithelia was observed (IC_50_ > 10 μM, Figure 1A). A similar lack of proliferative change was observed with PC3 and LNCaP PCa cells (data not shown). In contrast, an IC_50_ near 0.1 μM was observed with primary human PCa associated fibroblasts (CAF), with a significant down regulation of MTT activity (p value ≤ 0.001 Figure 1B). Both mouse bone marrow derived fibroblasts and macrophages were exquisitely sensitive to cabozantinib (p value ≤ 0.0001, Figure 1C, 1D). Thus, the anti-proliferative effect of cabozantinib was two orders of magnitude greater on the host cells compared to PCa epithelia, suggesting that cabozantinib has a differential effect on prostate tumors and the host.

**Figure 2:**
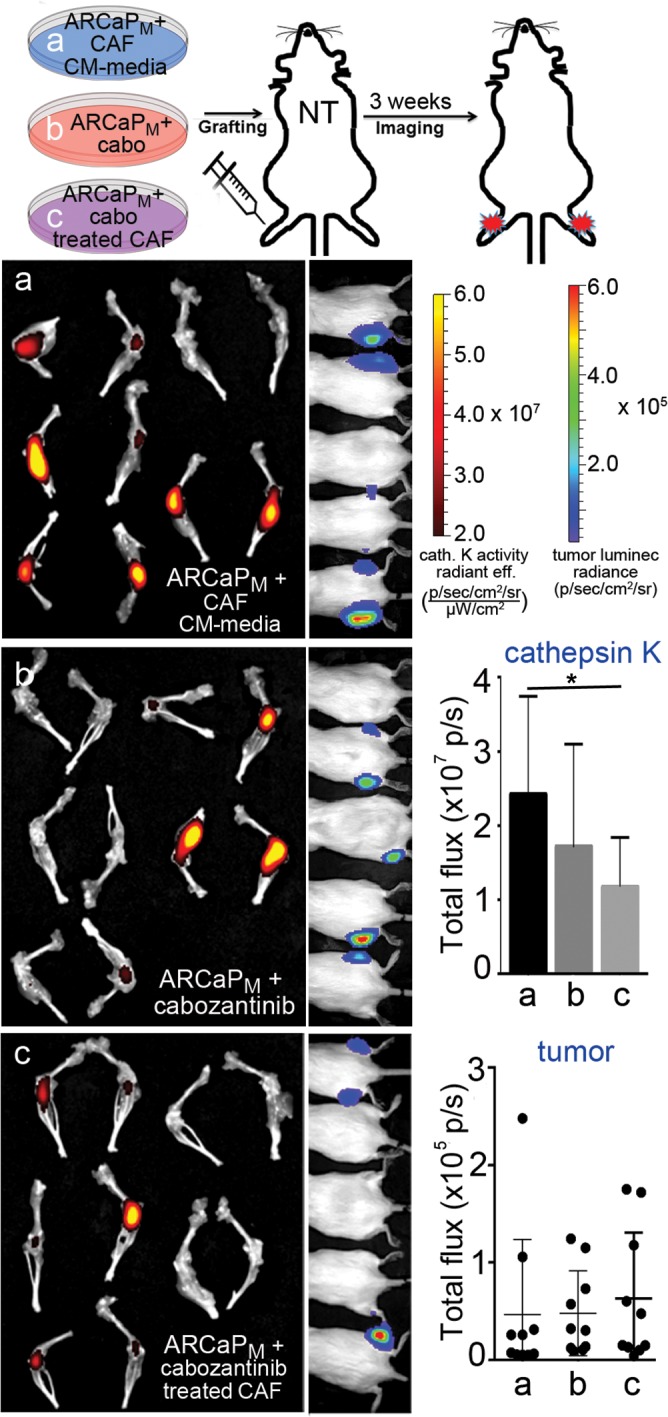
PCa cells pre-treated with conditioned medium from cabozantinib-exposed CAFs show reduced bone remodeling ability In this study cultured human CAF or ARCaP_M_ were treated with cabozantinib, but the mice were not treated. Tumor bioluminescence and osteoclast-associated cathepsin K activity imaging was performed on mouse tibiae inoculated with **(a)** ARCaP_M_-Luc cells treated with CAF conditioned media, **(b)** ARCaP_M_–Luc cells directly treated with cabozantinib, and **(c)** ARCaP_M_-Luc cells treated with conditioned media from CAFs exposed to cabozantinib. Corresponding quantification of cathepsin K activity by near infrared imaging and bioluminescence imaging is graphed. Statistical significance was determined using one-way ANOVA analysis with Dunnett's multiple comparisons test (^*^p value ≤ 0.05).

**Figure 3:**
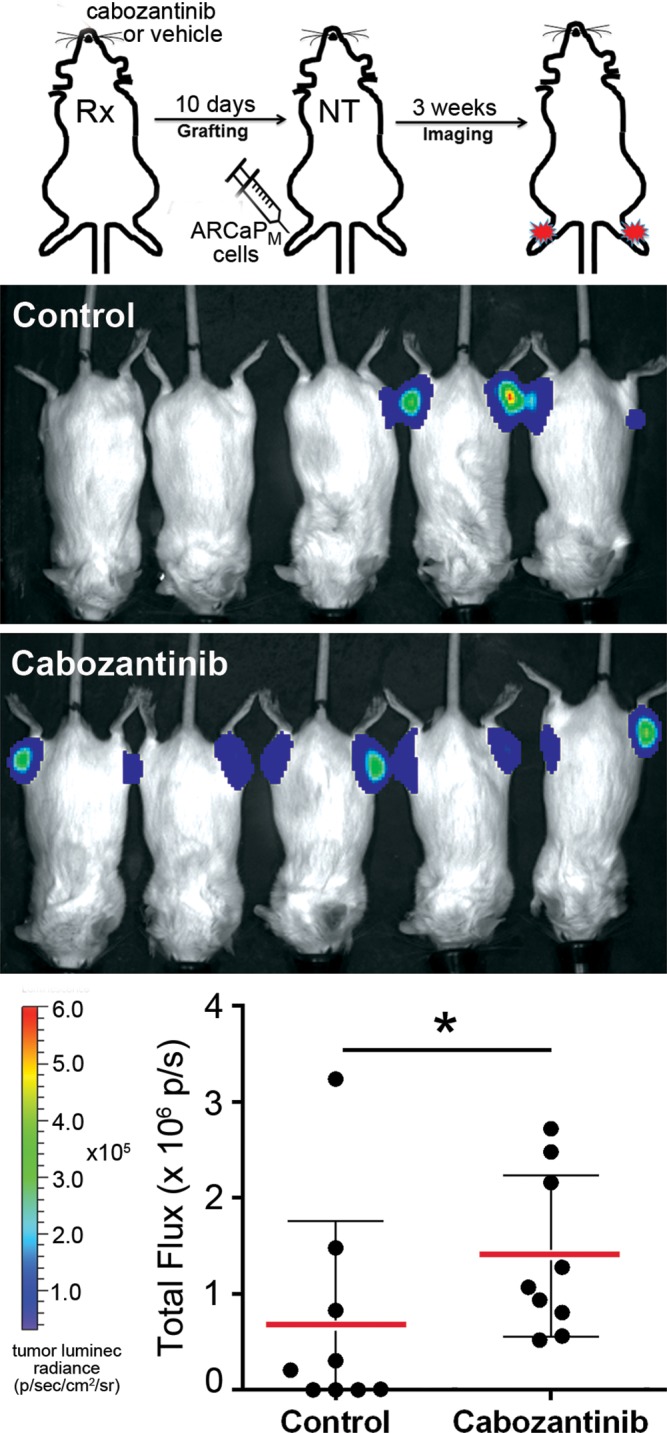
Cabozantinib pre-treated mice display increased efficiency of tumor uptake Schematic illustration of the experimental design (top) shows intra-tibial inoculation of ARCaP_M_-Luc cells in mice pre-treated with cabozantinib (Rx). The mice are not treated (NT) following introduction of tumor cells. Luciferase imaging shows tumor uptake at 4 weeks. A significant increase of tumor burden was observed in the cabozantinib-pretreated group compared to the control group (^*^p value ≤ 0.05).

The relative impact of cabozantinib on the viability of prostatic and bone marrow-derived fibroblasts supported the exploration of its effect on fibroblast-mediated paracrine activity. ARCaP_M_ cells were pre-treated *ex vivo* with 1) standard propagation media containing cabozantinib, 2) conditioned media (CM) from prostatic CAFs for two passages, or 3) CM from CAFs that were treated with cabozantinib. Utilizing the intra-tibial mouse model, the PCa epithelia in the three experimental arms were subsequently injected into the tibiae of beige-SCID mice to assay their ability to grow in the bone microenvironment (Figure 2). Importantly, in this set of experiments the mouse host was not treated with cabozantinib. Tumor expansion was monitored by the luciferase activity of ARCaP_M_-Luc cells, while osteoclast activity was visualized by osteoclast-cathepsin K activity. We found no significant difference on osteoclastic activity in mice injected with ARCaP_M_ cells treated with cabozantinib or those that were pre-treated with CAF-CM (Figure 2). However, there was a significant decrease in cathepsin K activity in ARCaP_M_ cells incubated with CM from cabozantinib pre-treated CAF compared to ARCaP_M_ cells incubated with CM from untreated CAF. Of note, the mean tumor size remained unaffected in all three conditions. Our findings are in congruence with a recent report that investigated the effect of cabozantinib on the bone microenvironment, and found that non-cytotoxic doses of cabozantinib significantly inhibited the differentiation of monocyte-derived primary osteoclasts obtained from healthy human donors [20]. In parallel, to assess paracrine effect of cabozantinib on PCa visceral metastasis, we utilized the intra-splenic injection model. ARCaP_M_ cells were incubated with CM from CAFs that were pre-treated with either cabozantinib or vehicle. We found that while every mouse injected with ARCaP_M_ cells, pre-treated with CM from vehicle-treated-CAFs developed tumors, no tumors were detected in mice that were injected with ARCaP_M_ cells, pre-treated with CM from cabozantinib-treated CAFs (Supplementary Figure 1). These findings suggested that in the context of the bone microenvironment, the paracrine effect of cabozantinib treatment inhibited the bone turnover of PCa lesions, but did not necessarily affect the tumor expansion. Although the bone tumor volume was not changed significantly, the osteoclast activity - which plays a critical role in tumor expansion within the PCa bone metastatic microenvironment - was significantly altered. It has been established by multiple investigators that during the progression of PCa bone metastasis, the expansion of the tumor in the bone is dependent on its unique environment and the cancer cells that colonize and expand in it [21–23]. On the other hand, in the visceral metastatic setting of PCa, the paracrine impact of cabozantinib was tumor inhibitory. The pro-tumorigenic effect of cabozantinib in the bone microenvironment juxtaposed with its minimal effect on tumor growth warranted further investigation of the host response to the drug. We therefore pre-treated mice with cabozantinib or vehicle for 10 days prior to inoculating them with ARCaP_M_ PCa epithelia. Following either intra-tibial or intra-splenic injection of mice, we found that the luciferase-expressing ARCaP_M_ cells expanded significantly more in the hosts pre-treated with cabozantinib compared with vehicle (p value < 0.05; Figure 3, Supplementary Figure 2). Of note, in these studies, the PCa cells were not treated with cabozantinib. The fact that treating the host with cabozantinib resulted in tumor expansion, further pointed to a pro-tumorigenic reprogramming of the microenvironment by cabozantinib.

### Cabozantinib affects macrophage polarity

**Figure 4:**
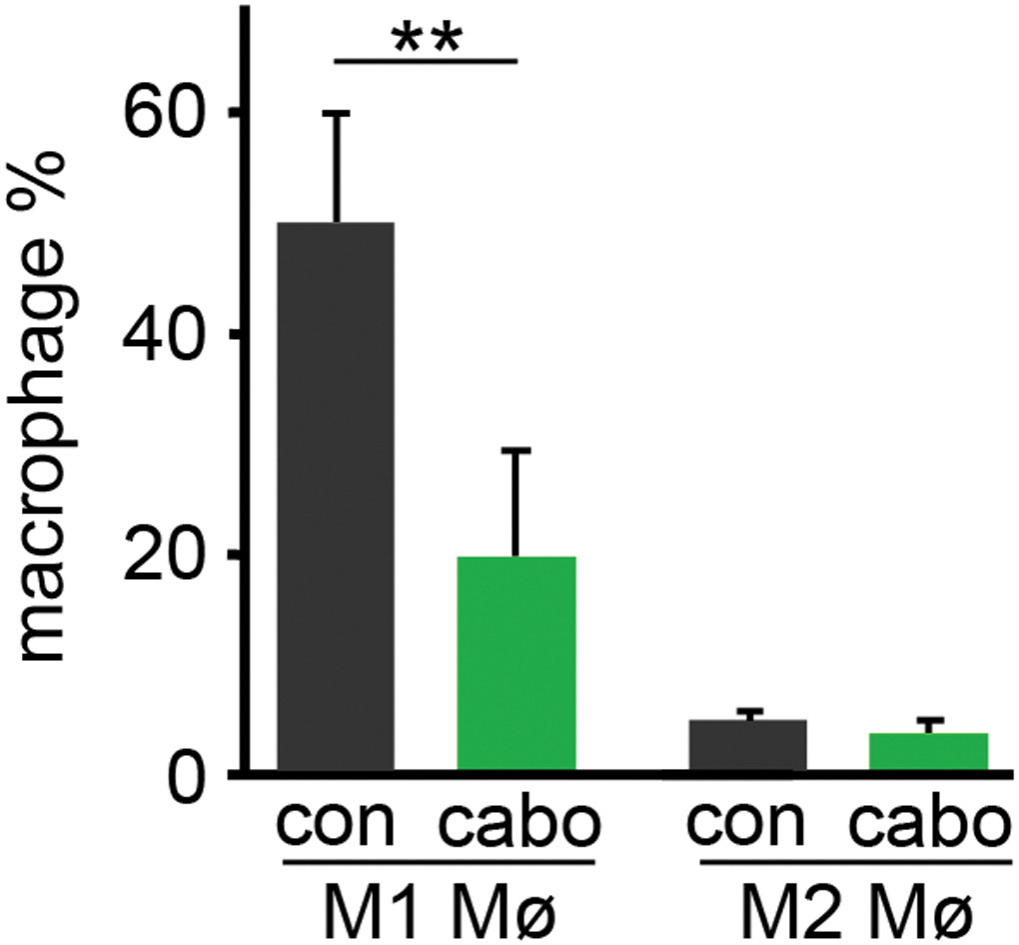
Cabozantinib regulate the polarization of macrophage recruited to tumors The macrophages extracted from tumors expanded in mice pretreated with vehicle or cabozantinib were analyzed by FACS. Cabozantinib significantly decreased the population of M1 macrophage without effecting the M2 macrophage, compared to vehicle treated mice. M1 and M2 polarity of F4/80+ macrophage was measured by FACS for F4/80+/MHCII+ (M1) and F4/80+/CD206+ (M2) (^**^p value ≤ 0.01).

**Figure 5:**
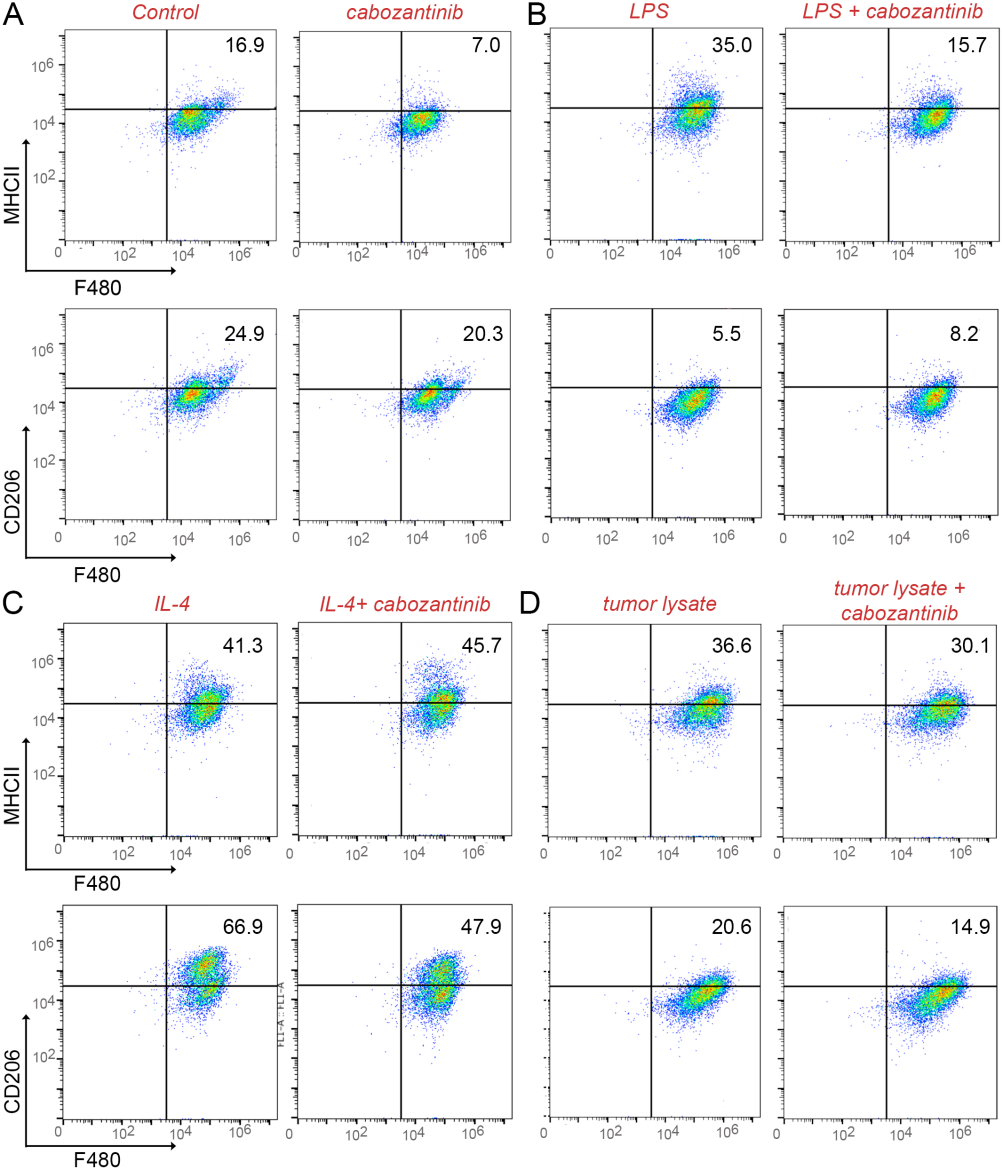
Cabozantinib reduces M1 macrophage populations Thioglycollate-induced, activated macrophages were cultured with or without lipopolysaccharide (LPS) or interleukin-4 (IL-4) or tumor lysate. **(A)** The addition of cabozantinib induced changes F4/80+/MHCII+ and F4/80+/CD206+ macrophage populations were measured by FACS. **(B)** LPS stimulated M1 population was impacted by cabozantinib treatment. **(C** and **D)** Cabozantinib effects on the IL-4-stimulated and tumor lysate-stimulated macrophage populations were tested, respectively.

To better understand the effects of cabozantinib on macrophage, splenic macrophages were analyzed in mice inoculated with ARCaP_M_ cells, and treated with cabozantinib or vehicle, as in the previous study. Interestingly, cabozantinib significantly decreased the M1 macrophage population (F4/80+, MHCII+; p value ≤ 0.05), yet had little effect on M2 macrophages (F4/80+, CD206+), compared with vehicle treated mice (Figure 4, Supplementary Figure 3). To further characterize the effect of cabozantinib on the macrophages, thioglycollate induced peritoneal macrophage from immuno-competent C57BL/6 mice were isolated. Macrophages were subjected to cabozantinib or vehicle treatment *ex vivo*. In agreement with the *in vivo* results, *ex vivo* treatment with cabozantinib demonstrated similar M1 macrophage population reduction, with the M2 macrophage population largely unaffected (Figure 5A). Cabozantinib treatment further down regulated LPS stimulated M1 macrophage population, without affecting the M2 macrophages under the same conditions (Figure 5B). When IL-4 was used to potentiate M2 macrophage polarization, the addition of cabozantinib reduced the M2 population with little effect on the M1 population (Figure 5C). Further, incubation of macrophages with tumor lysate and cabozantinib resulted in the reduction of both M1 and M2 populations, compared to the tumor lysate alone (Figure 5D). These effects of cabozantinib on the M1 macrophages were tested in mice bearing PC3 tumor xenografts. Similarly, we found that in these tumors, cabozantinib treatment significantly decreased the population of F4/80 and MHCII double-positive M1 macrophages compared to the control group (p value ≤ 0.01 Supplementary Figure 4). Together, cabozantinib was found to be a down-regulator of tumor-naïve M1 macrophages.

### Cabozantinib impacts macrophage differentiation in metastatic CRPC patients

**Figure 6:**
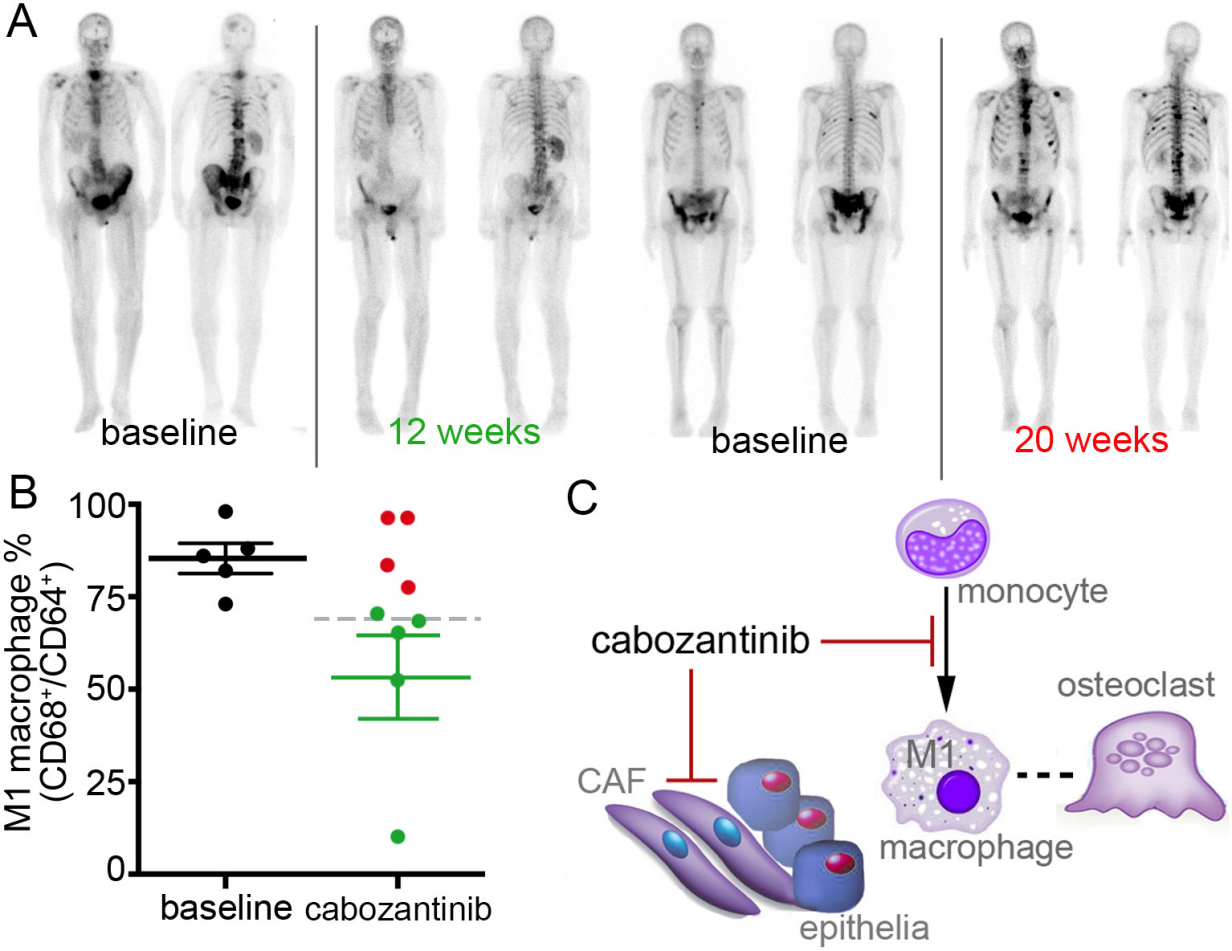
Monocyte-derived M1 macrophages can serve as a biomarker for cabozantinib efficacy in PCa bone metastasis **(A)** An example of Tc-99 bone scans that improved with cabozantinib treatment compared to untreated. Also, an example of Tc-99 bone scans that progressed with cabozantinib treatment compared to untreated. **(B)** Peripheral blood monocytes isolatedfrom patients (Table 1) were cultured with M-CSF and analyzed by FACS. The CD68+/CD64+ double positive M1 macrophage in all subjects prior to cabozantinib treatment was 85% (+/− 9.1 SD, black dots). M1 macrophage differentiation status in patients having cabozantinib-associated improvement of Tc-99 bone scans (p value = 0.03, green mean line and dots). Patients with no improvement in bone scans are in red. The gray dashed line depicts the overall mean of all subjects given cabozantinib. **(C)** Cabozantinib can act on the cells of tumor microenvironment. Cabozantinib treatment of prostatic CAF reduced bone turnover. Monocytes reprogramed by cabozantinib to inhibit M1 macrophage polarization can be a surrogate marker for osteoclast activity.

Since our mouse studies pointed to a cabozantinib-mediated effect on macrophage polarity, we next explored the potential effect of cabozantinib on macrophage differentiation in advanced metastatic CRPC patients. We examined monocytes from serial buffy coat samples of mCRPC patients before and during cabozantinib administration (Table 1). Patients were selected on the basis of their clinical and radiographic response to cabozantinib therapy as determined by conventional CT and Tc-99 radionuclide scan. Of the 9 patients, 5 had an improvement in Tc-99 radionuclide scan while on cabozantinib therapy (Table 1). Changes in serum PSA concentration, total macrophage phenotype, and circulating tumor cell number were not predictive of responses measured in metastatic lesions. To characterize the effect of cabozantinib on monocytes, circulating monocytes from these patients were differentiated into macrophages *ex vivo*. In this analysis, we focused on the M1 macrophage differentiation status of the selected patients (Table 1). The CD68+/CD64+ double positive M1 macrophage in all subjects prior to cabozantinib treatment was 85% (+/− 9.1 SD). Strikingly, for those patients that showed improvement on bone scans during cabozantinib therapy, monocyte-M1 macrophage differentiation was significantly down regulated from pre-treatment levels (p value = 0.03, Figure 6 and Table 1). Further, the patients that had monocyte-M1 macrophage differentiation similar to pre-treatment levels, had no bone scan improvement while on cabozantinib treatment (Figure 6 and Table 1). In all cases, the M2-type macrophage population was largely unchanged by cabozantinib treatment (data not shown). Of note, these changes in macrophage polarity did not associate with responses in visceral lesions or those that had no change in bone lesions. We noticed that in the data set there was an outlier M1 macrophage status value. However, even in the absence of the outlier patient, the reduced M1 macrophage status predicted a significant difference between the bone-responsive and non-responsive patients (p value = 0.0085).

**Table 1:** Clinical correlates of the cabozantinib treated patients used to assess monocyte-derived macrophage polarization reprograming

	Baseline blood work		1°	Weeks	PBMC derived M1-Mø	Blood work at imaging		Imaging at the time of PBMC assessment	
Patient	PSA	CTC	Rx	on cabo	Fraction on cabozantinib	PSA	CTC	Bone	Soft tissue
A	<0.1	5	M	68	77	<0.1	15	Stable	Progressed
B	3826	131	R	20	52	16855	905	Improved	Progression
C	11	25	P	16	68	11.2	0	Improved	Improved
D	5.2	106	M	28	83	9.6	6	Stable	Stable
F	1.0	0	M	20	97	1.1	1	Progression	Stable
G^*^	3.1	146	M	2	65	N/A	N/A	N/A	N/A
H	3.2	74	M	4	9.6	15.5	47	CR	Progressed
I	50.7	43	M	2	70	242.1	25	Improved	Progressed
J	0.6	3	M	2	96	3.0	9	Mixed Res	Progressed

M = metastatic at presentation.

P = prostatectomy.

R = radiation ablation.

CR = complete response.

^*^Died before imaging could be done.

The baseline and weeks on cabozantinib (cabo) treatment are indicated with associated PSA, and circulating tumor cell (CTC) values. The patient status prior treatment and nature of tumor progression in terms of soft tissue and bone metastasis status at the time of peripheral blood monocytic cells (PBMC) assessment is indicated. The percentage of M1 polarization of macrophage (Mø), derived from circulating monocytes, was determined by CD68, CD64 expression.

## DISCUSSION

There is a clear need to identify therapeutics that address both cancer epithelia and its microenvironment in PCa and other malignancies. A growing body of evidence underscores the impact of kinase inhibitors on the immune microenvironment surrounding the tumor cells [24–28]. In this study, we present the first evidence of differential cabozantinib effects on PCa epithelia and the surrounding microenvironment. Although, the immune modulatory role of cabozantinib has previously been attributed to reduced regulatory T cell function [29], myeloid-derived suppressor cells (MDSCs) [30] and neutrophils [31, 32]. Our study identified a correlation between macrophage polarization and a positive therapeutic response in cabozantinib-treated advanced mCRPC patients. Further, our finding that cabozantinib provoked a macrophage response as part of its mechanism of action has potential implications for the use of this drug in treatment modalities that may compromise macrophage function such as taxane therapy. Conventional taxane therapy is known to deplete M2 macrophage and expand M1 macrophage [33]. Our data showed that a relatively short duration of cabozantinib treatment was sufficient to induce monocyte reprogramming, a response that we found could predict a favorable bone response often associated with reduced bone pain.

Although PSA concentration and CTC conversion have not been predictive of PCa patient progression in the past (Table 1) [14], an early (i.e. within 2-4 weeks) minimally invasive test for cabozantinib reactivity on bone metastasis could be useful in determining therapeutic efficacy well before a patient incurs excessive toxicity and/or delays in alternative therapeutic strategies. In this context, our finding that cabozantinib-treated cancer associated fibroblasts and macrophages affect bone turnover is in agreement with a recent report showing that cabozantinib inhibits the differentiation of monocyte-derived primary human osteoclasts [20]. Our observations in mice and in patients support that monocyte-derived M1 macrophage polarization may be a surrogate to osteoclast activity in cabozantinib-treated mCRPC patients.

Multiple investigators have reported that cabozantinib suppresses both PCa-induced metastatic bone destruction and tumor progression in visceral tissues [9, 34, 35]. However, overall survival benefit from the drug could be achieved by improved patient selection based on the effects cabozantinib has on the microenvironment. Previous reports support a potential cytostatic role for cabozantinib on PCa epithelia at supra-physiologic doses [9, 34, 35]. Along similar lines, we found that direct measures of epithelial proliferation were largely unchanged by physiologically relevant doses of cabozantinib. We do not claim that the M1 macrophage down regulation was the reason for increased tumor expansion. In congruity with previous reports that prostatic fibroblasts have a role in bone engraftment [36], we found that cabozantinib can abrogate such paracrine mediators in prostatic fibroblasts (Figure 2). Cabozantinib has reported anti-angiogenic effects, however, the potential vascular abrogative role of cabozantinib was excluded in our models since we treated either the host or the tumor cells independently, and found that the tumor either expanded or had no change. Our results instead support cabozantinib-induced reprogramming of the monocytes and PCa epithelia independent of it anti-angiogenic effects. Some of the cabozantinib-induced macrophage polarity changes were observed in immunocompromised mice. Nonetheless, the results from the patient samples showed that an improvement on bone scans during cabozantinib therapy is associated with a down regulation of monocyte-M1 macrophage differentiation, as observed in mice. Conversely, the patients that did not show an improvement on the bone scan during cabozantinib treatment correspondingly did not display a down regulation in the monocyte-M1 macrophage differentiation. Regardless, the results in Figure 6 reveals the drop of M1 macrophage population which supports patient response. Importantly, our work sets the stage for trying to understand the complex role of M1 macrophage in bone metastases and reprogramming as a result of cabozantinib treatment. Our finding that cabozantinib effects macrophage polarization as part of its mechanism of action has broader implications as a biomarker for patient stratification, as well as helping decide the best treatment regimen. As patient heterogeneity cannot be addressed by the small patient numbers available to us in this study, a larger prospective study is needed.

## MATERIALS AND METHODS

### Reagents and cytokines

Recombinant murine IFN-γ, granulocyte-macrophage colony-stimulating factor (GM-CSF), granulocyte colony-stimulating factor (G-CSF), M-CSF, SCF, IL-4, IL-6 were obtained from PeproTech (Rocky Hill, NJ). Human recombinant M-CSF was obtained from R&D Systems (Minneapolis, MN). Cabozantinib was provided by Exelixis Inc. (South San Francisco, CA) through the NIH/NCI Cancer Therapy Evaluation Program (CTEP).

### Clinical samples, annotation, isolation and culture of human monocytes

Blood samples were obtained from mCRPC patients treated with cabozantinib after documented informed consent was obtained for an institutional review board-approved study (NCT01834651 CSMC IRB Pro00030191). Response to therapy was determined by review of radiographic studies. Buffy coats were frozen and thawed once. Monocytes were enriched from PBMC by a standard Ficoll gradient (GE Healthcare Lifesciences) and the attached cells were cultured. Macrophages were generated by supplementation of 20 ng/ml hrM-CSF and analyzed by flow cytometry.

### Macrophage flow cytometric analysis

After isolation, the peritoneal- and bone marrow-derived macrophages from mice were culture and analyzed by FACS using the following antibodies: F4/80-APC, CD206-Alexa Fluor 488, CD80-PE, anti-mouse CD86-PE, CD11c-APC (eBioscience). Human macrophages were analyzed following Fc receptor blocking with Human TruStain FcX™ (Biolegend), using antibodies to CD64-APC, CD163-PE-Cyanine7, CD206-Alexa Fluor 488, and CD68-PE (eBioscience). Data were collected by LSR II flow cytometry (BD) and analyzed with FlowJo software (FlowJo Enterprises, Ashland OR).

### Cell culture

Primary mouse prostate stromal cell cultures were generated from 6–8-week-old C57BL/6 mice as previously described [37]. Human prostate stromal cells were similarly developed from fresh human prostatectomy tissues. All tissue procurement and utilization was conducted under institutional review board protocols. Malignancy was identified by pathologic review after hematoxylin and eosin staining of frozen sections. Human prostate cancer cells, ARCaP_M_ were cultured in T-medium (Invitrogen) supplemented with 5% fetal bovine serum. The PC3 and Myc-CaP cells were cultured in DMEM medium supplemented with 10% fetal bovine serum and 1% penicillin/streptomycin at 37°C, 5% CO2. Cell viability was assessed using MTT assay as indicated by the manufacturer (ThermoFisher, Canoga Park, CA). Assays were performed in replicates of five.

### Mouse studies

Procedures and animal experiments were approved by the Institutional Animal Care and Use Committee at Cedars-Sinai Medical Center.

Intratibial tumor expansion studies were performed in adult severe combined immuno-deficient (beige SCID) male mice (4- to 10-week-old male mice) obtained from the Envigo (Indianapolis, IN, USA) [36]. ARCaP_M_ cells with a firefly luciferase reporter construct were incubated for 72 hours with or without conditioned media from primary cultured prostatic fibroblasts treated with cabozantinib or DMSO. Tumor growth was monitored by bioluminescence imaging.

### Generation of peritoneal macrophages

Thioglycollate-elicited peritoneal exudate cells were collected via peritoneal lavage 4 days after a 2-ml injection of 3% thioglycollate (Sigma) broth intraperitoneally and were cultured in tissue culture-treated plates (1 × 10^6^/ml) at 37 °C and 5% CO_2_ in 10% fetal calf serum RPMI 1640, 50 μM 2-ME, 0.5 mM sodium pyruvate, 10 mM HEPES buffer, 50 unit/ml penicillin, 50 μg/ml streptomycin, and 2 mM L-glutamine. For purification of thioglycollate-elicited peritoneal macrophage, peritoneal exudate cells were allowed to adhere for 2 hr, after which nonadherent cells were washed off to achieve a >95% purity of macrophage. For flow cytometry assay, peritoneal exudate cells were treated and cultured in Costar low adherence culture plates.

### Generation of mouse bone marrow-derived macrophages

Bone marrow-derived macrophages were generated as previously described with some modifications [38]. In brief, bone marrow was harvested from the femurs and tibiae of 7-week old C57BL/6 wild type and filtered with 40 μM pore nylon cell strainers (BD Biosciences, Franklin Lakes NJ). The cells were cultured in RPMI 1640, 10% (v/v) heat-inactivated FBS, antibiotics, and 100 ng/ml recombinant murine M-CSF.

## SUPPLEMENTARY FIGURES



## References

[1] Merlo LM, Pepper JW, Reid BJ, Maley CC (2006). Cancer as an evolutionary and ecological process. Nat Rev Cancer.

[2] Yakes FM, Chen J, Tan J, Yamaguchi K, Shi Y, Yu P, Qian F, Chu F, Bentzien F, Cancilla B, Orf J, You A, Laird AD (2011). Cabozantinib (XL184), a novel MET and VEGFR2 inhibitor, simultaneously suppresses metastasis, angiogenesis, and tumor growth. Mol Cancer Ther.

[3] Kurzrock R, Sherman SI, Ball DW, Forastiere AA, Cohen RB, Mehra R, Pfister DG, Cohen EE, Janisch L, Nauling F, Hong DS, Ng CS, Ye L (2011). Activity of XL184 (Cabozantinib), an oral tyrosine kinase inhibitor, in patients with medullary thyroid cancer. J Clin Oncol.

[4] Zhang T, Park SE, Hong C, George DJ (2016). Cabozantinib in genitourinary malignancies. Future Oncol.

[5] Neal JW, Dahlberg SE, Wakelee HA, Aisner SC, Bowden M, Huang Y, Carbone DP, Gerstner GJ, Lerner RE, Rubin JL, Owonikoko TK, Stella PJ, Steen PD (2016). Erlotinib, cabozantinib, or erlotinib plus cabozantinib as second-line or third-line treatment of patients with EGFR wild-type advanced non-small-cell lung cancer (ECOG-ACRIN 1512): a randomised, controlled, open-label, multicentre, phase 2 trial. Lancet Oncol.

[6] Singh H, Brave M, Beaver JA, Cheng J, Tang S, Zahalka E, Palmby TR, Venugopal R, Song P, Liu Q, Liu C, Yu J, Chen XH (2016). Food and Drug Administration Approval: cabozantinib for treatment of advanced renal cell carcinoma. Clin Cancer Res.

[7] Tolaney SM, Ziehr DR, Guo H, Ng MR, Barry WT, Higgins MJ, Isakoff SJ, Brock JE, Ivanova EV, Paweletz CP, Demeo MK, Ramaiya NH, Overmoyer BA (2016). Phase II and biomarker study of cabozantinib in metastatic triple-negative breast cancer patients. Oncologist.

[8] Graham TJ, Box G, Tunariu N, Crespo M, Spinks TJ, Miranda S, Attard G, de Bono J, Eccles SA, Davies FE, Robinson SP (2014). Preclinical evaluation of imaging biomarkers for prostate cancer bone metastasis and response to cabozantinib. J Natl Cancer Inst.

[9] Dai J, Zhang H, Karatsinides A, Keller JM, Kozloff KM, Aftab DT, Schimmoller F, Keller ET (2014). Cabozantinib inhibits prostate cancer growth and prevents tumor-induced bone lesions. Clin Cancer Res.

[10] Basch E, Autio KA, Smith MR, Bennett AV, Weitzman AL, Scheffold C, Sweeney C, Rathkopf DE, Smith DC, George DJ, Higano CS, Harzstark AL, Sartor AO (2015). Effects of cabozantinib on pain and narcotic use in patients with castration-resistant prostate cancer: results from a phase 2 nonrandomized expansion cohort. Eur Urol.

[11] Saylor PJ, Lee RJ, Smith MR (2011). Emerging therapies to prevent skeletal morbidity in men with prostate cancer. J Clin Oncol.

[12] Smith DC, Smith MR, Sweeney C, Elfiky AA, Logothetis C, Corn PG, Vogelzang NJ, Small EJ, Harzstark AL, Gordon MS, Vaishampayan UN, Haas NB, Spira AI (2013). Cabozantinib in patients with advanced prostate cancer: results of a phase II randomized discontinuation trial. J Clin Oncol.

[13] Smith MR, Sweeney CJ, Corn PG, Rathkopf DE, Smith DC, Hussain M, George DJ, Higano CS, Harzstark AL, Sartor AO, Vogelzang NJ, Gordon MS, de Bono JS (2014). Cabozantinib in chemotherapy-pretreated metastatic castration-resistant prostate cancer: results of a phase II nonrandomized expansion study. J Clin Oncol.

[14] Smith M, De Bono J, Sternberg C, Le Moulec S, Oudard S, De Giorgi U, Krainer M, Bergman A, Hoelzer W, De Wit R, Bogemann M, Saad F, Cruciani G (2016). Phase III study of cabozantinib in previously treated metastatic castration-resistant prostate cancer: COMET-1. J Clin Oncol.

[15] Choueiri TK, Escudier B, Powles T, Mainwaring PN, Rini BI, Donskov F, Hammers H, Hutson TE, Lee JL, Peltola K, Roth BJ, Bjarnason GA, Geczi L (2015). Cabozantinib versus everolimus in advanced renal-cell carcinoma. N Engl J Med.

[16] Santini D, Tonini G (2016). Treatment of advanced renal-cell carcinoma. N Engl J Med.

[17] Motzer RJ, Escudier B, Choueiri TK (2016). Treatment of advanced renal-cell carcinoma. N Engl J Med.

[18] Ott PA, Adams S (2011). Small-molecule protein kinase inhibitors and their effects on the immune system: implications for cancer treatment. Immunotherapy.

[19] Sica A, Mantovani A (2012). Macrophage plasticity and polarization: in vivo veritas. J Clin Invest.

[20] Fioramonti M, Santini D, Iuliani M, Ribelli G, Manca P, Papapietro N, Spiezia F, Vincenzi B, Denaro V, Russo A, Tonini G, Pantano F (2017). Cabozantinib targets bone microenvironment modulating human osteoclast and osteoblast functions. Oncotarget.

[21] Zhang J, Dai J, Qi Y, Lin DL, Smith P, Strayhorn C, Mizokami A, Fu Z, Westman J, Keller ET (2001). Osteoprotegerin inhibits prostate cancer-induced osteoclastogenesis and prevents prostate tumor growth in the bone. J Clin Invest.

[22] Mundy GR (2002). Metastasis to bone: causes, consequences and therapeutic opportunities. Nat Rev Cancer.

[23] Weilbaecher KN, Guise TA, McCauley LK (2011). Cancer to bone: a fatal attraction. Nat Rev Cancer.

[24] Steinberg SM, Zhang P, Malik BT, Boni A, Shabaneh TB, Byrne KT, Mullins DW, Brinckerhoff CE, Ernstoff MS, Bosenberg MW, Turk MJ (2014). BRAF inhibition alleviates immune suppression in murine autochthonous melanoma. Cancer Immunol Res.

[25] Sottile R, Pangigadde PN, Tan T, Anichini A, Sabbatino F, Trecroci F, Favoino E, Orgiano L, Roberts J, Ferrone S, Karre K, Colucci F, Carbone E (2016). HLA class I downregulation is associated with enhanced NK-cell killing of melanoma cells with acquired drug resistance to BRAF inhibitors. Eur J Immunol.

[26] Vella LJ, Pasam A, Dimopoulos N, Andrews M, Knights A, Puaux AL, Louahed J, Chen W, Woods K, Cebon JS (2014). MEK inhibition, alone or in combination with BRAF inhibition, affects multiple functions of isolated normal human lymphocytes and dendritic cells. Cancer Immunol Res.

[27] Manzini C, Vene R, Cossu I, Gualco M, Zupo S, Dono M, Spagnolo F, Queirolo P, Moretta L, Mingari MC, Pietra G (2016). Cytokines can counteract the inhibitory effect of MEK-i on NK-cell function. Oncotarget.

[28] Hu-Lieskovan S, Mok S, Homet Moreno B, Tsoi J, Robert L, Goedert L, Pinheiro EM, Koya RC, Graeber TG, Comin-Anduix B, Ribas A (2015). Improved antitumor activity of immunotherapy with BRAF and MEK inhibitors in BRAF (V600E) melanoma. Sci Transl Med.

[29] Kwilas AR, Ardiani A, Donahue RN, Aftab DT, Hodge JW (2014). Dual effects of a targeted small-molecule inhibitor (cabozantinib) on immune-mediated killing of tumor cells and immune tumor microenvironment permissiveness when combined with a cancer vaccine. J Transl Med.

[30] Lu X, Horner JW, Paul E, Shang X, Troncoso P, Deng P, Jiang S, Chang Q, Spring DJ, Sharma P, Zebala JA, Maeda DY, Wang YA (2017). Effective combinatorial immunotherapy for castration-resistant prostate cancer. Nature.

[31] Patnaik A, Swanson KD, Csizmadia E, Solanki A, Landon-Brace N, Gehring MP, Helenius K, Olson BM, Pyzer AR, Wang LC, Elemento O, Novak J, Thornley TB (2017). Cabozantinib eradicates advanced murine prostate cancer by activating anti-tumor innate immunity. Cancer Discov.

[32] Sidaway P (2017). Prostate cancer: cabozantinib activates innate immunity. Nat Rev Urol.

[33] De Palma M, Lewis CE (2013). Macrophage regulation of tumor responses to anticancer therapies. Cancer Cell.

[34] Haider MT, Hunter KD, Robinson SP, Graham TJ, Corey E, Dear TN, Hughes R, Brown NJ, Holen I (2015). Rapid modification of the bone microenvironment following short-term treatment with Cabozantinib in vivo. Bone.

[35] Sennino B, Ishiguro-Oonuma T, Wei Y, Naylor RM, Williamson CW, Bhagwandin V, Tabruyn SP, You WK, Chapman HA, Christensen JG, Aftab DT, McDonald DM (2012). Suppression of tumor invasion and metastasis by concurrent inhibition of c-Met and VEGF signaling in pancreatic neuroendocrine tumors. Cancer Discov.

[36] Li X, Sterling JA, Fan KH, Vessella RL, Shyr Y, Hayward SW, Matrisian LM, Bhowmick NA (2012). Loss of TGF-beta responsiveness in prostate stromal cells alters chemokine levels and facilitates the development of mixed osteoblastic/osteolytic bone lesions. Mol Cancer Res.

[37] Kiskowski MA, Jackson RS, Banerjee J, Li X, Kang M, Iturregui JM, Franco OE, Hayward SW, Bhowmick NA (2011). Role for stromal heterogeneity in prostate tumorigenesis. Cancer Res.

[38] Manzanero S (2012). Generation of mouse bone marrow-derived macrophages. Methods Mol Biol.

