# Supplementary material for: Modulation of cabozantinib efficacy by the prostate tumor microenvironment
Source: Oncotarget. 2017 Sep 23;8(50):87891–902. doi: 10.18632/oncotarget.21248 (PMC5675680; doi:10.18632/oncotarget.21248)
Supplement: Supplementary file 1 [file oncotarget-08-87891-s001.pdf]

Modulation of cabozantinib efficacy by the prostate tumor microenvironment

SUPPLEMENTARY MATERIALS

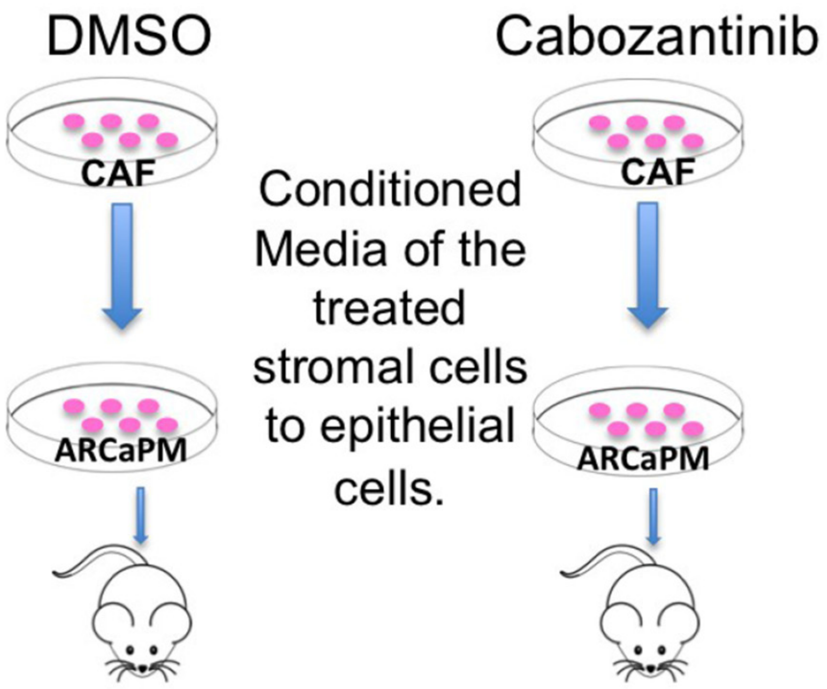

Group	No of animals with Metastasis
DMSO	4/5
Cabozantinib	0/6

Supplementary Figure 1: Visceral tumor formation demonstrates the paracrine role of cabozantinib on ARCaP$_M$ cells. Conditioned media from cabozantinib treated prostatic fibroblasts were incubated with ARCaP$_M$ cells grown in culture. Subsequently, the ARCaP$_M$ cells were introduced by intrasplenic injection into mice. The expansion of tumors in the mice were evaluated by bioluminescence and visual verification. The ARCaP$_M$ cells or host mice were never treated with cabozantinib.

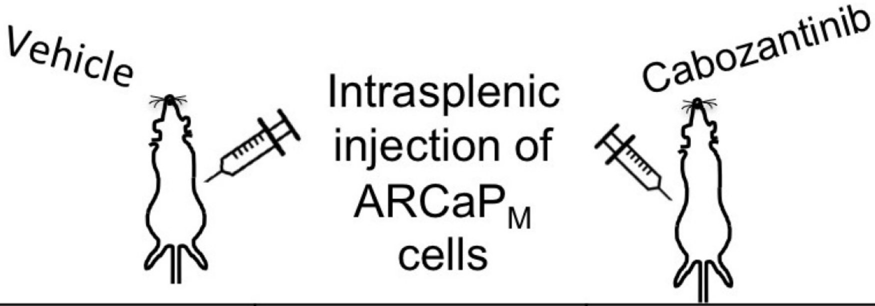

Group	Vehicle	Cabozantinib
No of animals with Metastasis	0/5	2/5

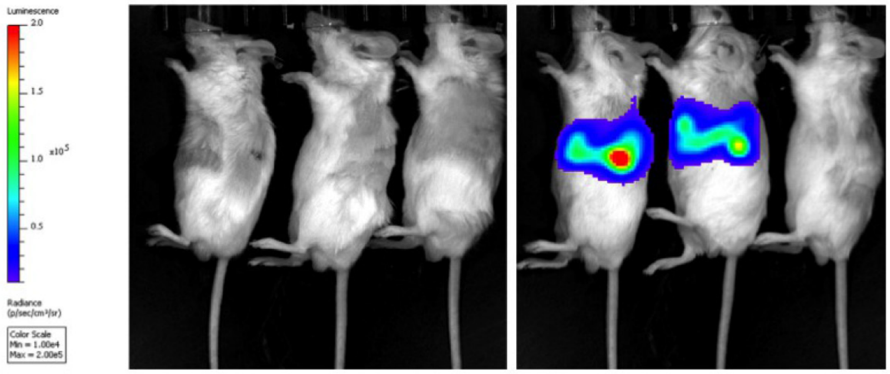

Supplementary Figure 2: Cabozantinib pre-treated mice have increased soft tissue tumor uptake. Luciferase imaging showing tumor uptake at 4 weeks following inter-splenic inoculation of 5×10^5 $ARCaP_M$-Luc cells. Briefly, $ARCaP_M$ cells were injected into the spleen of beige SCID mice with a 27-gauge needle. Bioluminescence imaging was used to monitor development of tumors. Image analysis was performed with Living Image software (PerkinElmer, Waltham, MA) by measurement of photon flux. A significant increase of tumor burden was observed in the cabozantinib pretreated mice group compared to control group.

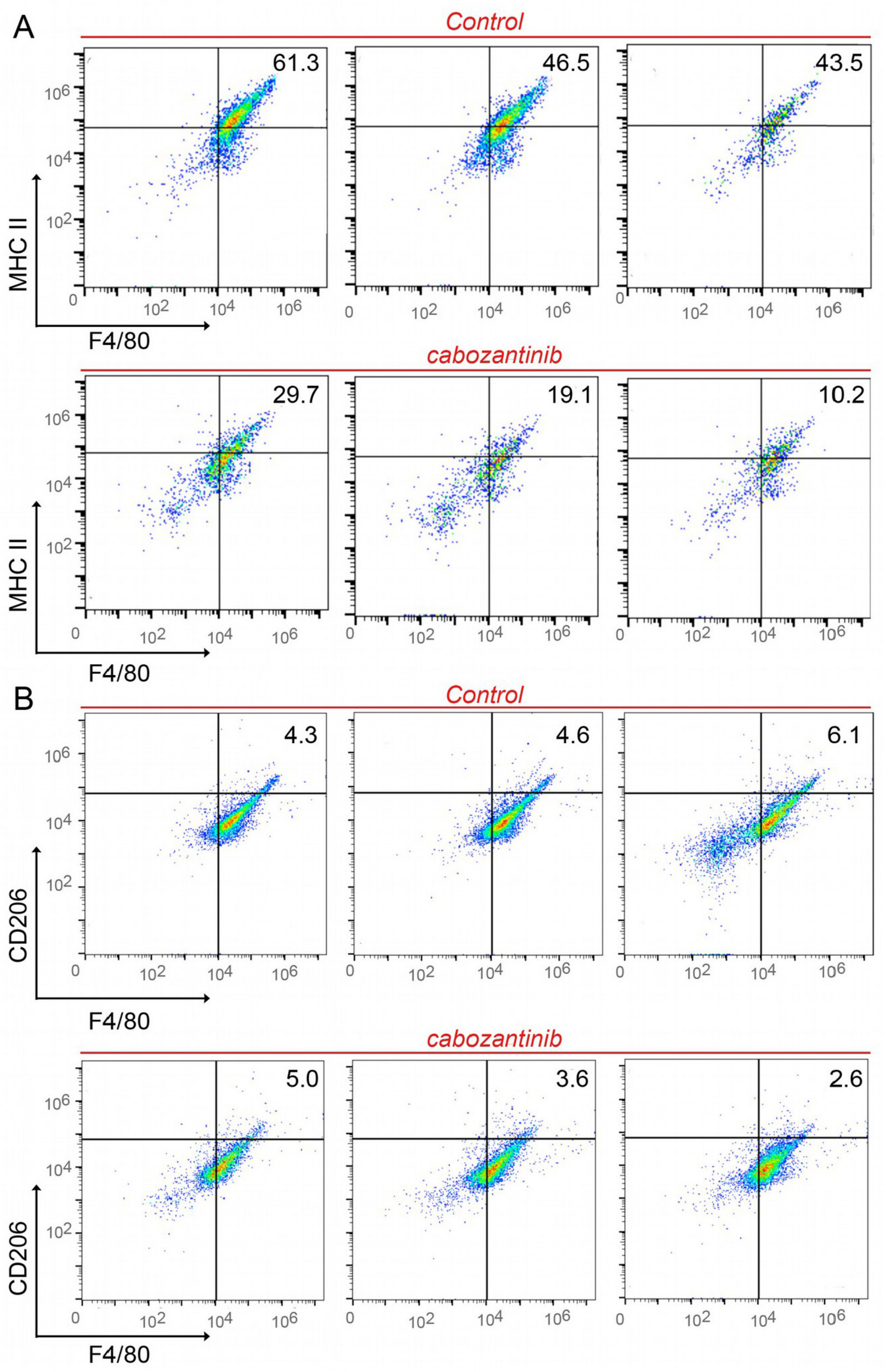

Supplementary Figure 3: Cabozantinib regulate the polarization of macrophage recruited to tumors. M1 and M2 polarity of F4/80+ macrophage were measured by the expression of MHCII and CD206, respectively.

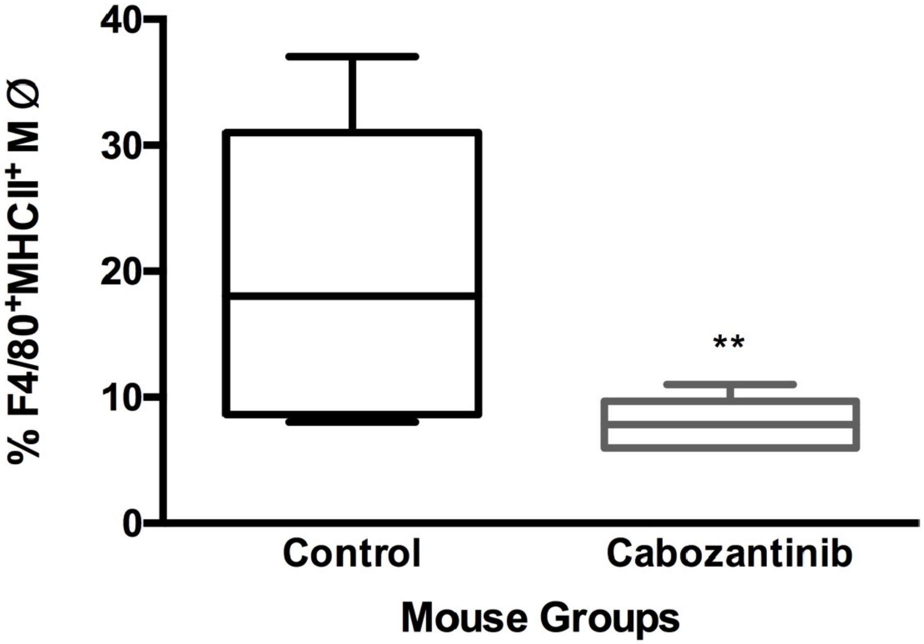

Supplementary Figure 4: Cabozantinib reduces M1 macrophage population *in vivo*. Reduction in the percentage of F4/80 and MHCII double positive macrophage population in the subcutaneous PC3 tumors due to cabozantinib treatment (**p value ≤ 0.01).
